# Changes in Alcoholic Beverage Choice and Risky Drinking among Adolescents in Europe 1999–2019

**DOI:** 10.3390/ijerph182010933

**Published:** 2021-10-18

**Authors:** Johanna K. Loy, Nicki-Nils Seitz, Elin K. Bye, Paul Dietze, Carolin Kilian, Jakob Manthey, Kirsimarja Raitasalo, Renate Soellner, Björn Trolldal, Jukka Törrönen, Ludwig Kraus

**Affiliations:** 1 IFT Institut für Therapieforschung, 80804 München, Germany; loy@ift.de (J.K.L.); seitz@ift.de (N.-N.S.); 2 Department of Alcohol, Tobacco and Drugs, Norwegian Institute of Public Health, 0213 Oslo, Norway; ElinKristin.Bye@fhi.no; 3 National Drug Research Institute, Curtin University, Melbourne, VIC 3004, Australia; paul.dietze@burnet.edu.au; 4 Behaviours and Health Risks Program, Burnet Institute, Melbourne, VIC 3004, Australia; 5 Institute of Clinical Psychology and Psychotherapy, Technische Universität Dresden, Chemnitzer Straße 46, 01187 Dresden, Germany; carolin.kilian@mailbox.org (C.K.); jakobmanthey@snappyquest.org (J.M.); 6 Centre for Interdisciplinary Addiction Research, UKE Hamburg-Eppendorf, Martinistraße 52, 20246 Hamburg, Germany; 7 Department of Psychiatry, Medical Faculty, University of Leipzig, Semmelweisstraße 10, 04103 Leipzig, Germany; 8 Finnish Institute for Health and Welfare, Health and Well-Being Promotion Unit, 00271 Helsinki, Finland; kirsimarja.raitasalo@thl.fi; 9 Department of Psychology, University of Hildesheim, Universitätsplatz 1, 31141 Hildesheim, Germany; soellner@uni-hildesheim.de; 10 The Swedish Council for Information on Alcohol and other Drugs (CAN), 116 64 Stockholm, Sweden; bjorn.trolldal@can.se; 11 Department of Public Health Sciences, Centre for Social Research on Alcohol and Drugs, Stockholm University, 106 91 Stockholm, Sweden; jukka.torronen@su.se; 12 Institute of Psychology, ELTE, Eötvös Loránd University, 1053 Budapest, Hungary

**Keywords:** beverage proportions, temporal changes, drinking patterns, youth drinking, alcohol use

## Abstract

This paper explores trends in beverage preference in adolescents, identifies related regional differences, and examines cluster differences in key drinking measures. Data were obtained from the European School Survey Project on Alcohol and Other Drugs (ESPAD), covering 24 European countries between 1999 and 2019. Trends in the distribution of alcoholic beverages on the participants’ most recent drinking occasion were analysed by sex and country using fractional multinomial logit regression. Clusters of countries based on trends and predicted beverage proportions were compared regarding the prevalence of drinkers, mean alcohol volume and prevalence of heavy drinking. Four distinct clusters each among girls and boys emerged. Among girls, there was not one type of beverage that was preferred across clusters, but the proportion of cider/alcopops strongly increased over time in most clusters. Among boys, the proportion of beer decreased, but was dominant across time in all clusters. Only northern European countries formed a geographically defined region with the highest prevalence of heavy drinking and average alcohol volume in both genders. Adolescent beverage preferences are associated with mean alcohol volume and heavy drinking at a country-level. Future approaches to drinking cultures need to take subpopulations such as adolescents into account.

## 1. Introduction

Alcohol consumption is one of the most important risk factors for adverse health effects and mortality [1]. Adolescents are particularly vulnerable to alcohol-related brain damage and the acquisition of problematic drinking behaviours [2,3]. Beverage choice or preference are known to be associated with risky single-occasion drinking [4] or drinking to acute intoxication [5], mean alcohol intake [4,6], self-reported health status, age and sex [7,8,9,10]. While adolescents’ alcohol consumption is declining in many European countries, little is known about how this is expressed in terms of the distribution of the different types of beverages they consume on specific drinking occasions. In addition, little is known about how the distribution of different beverages relates to key measures of alcohol consumption, such as mean alcohol volume and heavy drinking on recent drinking occasions, or how this varies among adolescents across European countries.

There is evidence for an association between the preference for different types of alcoholic beverages and several aspects of alcohol consumption among adolescents. Indicators of risky drinking such as high mean alcohol volume and heavy use are related to beverage choice. Consumers of beer and spirits typically drink more and show higher rates of heavy use as compared to wine drinkers. A preference for wine, in contrast, is known to be associated with less risky drinking [4,5,7,9,11,12]. Similarly, it has been shown that beverage preference among adolescents is also associated with health behaviour beyond alcohol consumption, such as smoking or healthy diet [4,13,14]. Furthermore, consumers preferring beer and spirits and all three beverage types were not only found to be heavier drinkers but were also more likely to engage in delinquent behaviour [13] and experience alcohol-related violence [15].

Beverage choice is subject to temporal changes and differs between countries [8,16,17,18]. Studies on beverage preferences in the general population have seen countries classified as predominantly wine-, beer- and/or spirit-drinking countries, later named wine-, beer- and former spirit-countries [8,19,20]. It has been suggested that the term ‘former spirits cultures’ be replaced by ‘present beer and wine countries’ [21]. Different framings of drinking culture have been used to classify countries. Countries characterized by low abstinence rates and low levels of heavy drinking (typically Mediterranean) have previously been denoted as having a ‘wet’ drinking culture, where alcohol is mainly consumed with meals and drinking is part of everyday social life, usually practised at home and generally involving the consumption of wine [8,22,23,24]. Countries with high levels of intoxication, where wine consumption is traditionally less common and where beer and spirits are the preferred beverages, have been classified as having a ‘dry’ drinking culture. These drinking patterns have often been found in northern European countries [25,26]. Importantly, in countries with high levels of intoxication, rates of alcohol-attributable harms were reported to be more prevalent [27]. Due to an increasing homogenisation of drinking patterns in Europe, the dry/wet distinction is no longer considered applicable [17,28,29]. This homogenisation also applies to beverage preferences [16], meaning that the classification of countries as having wine-, beer- and (former) spirit-drinking cultures may also no longer apply [21,28,29]. However, the recent literature on drinking cultures suggests that beverage choice is a decisive factor when describing different drinking behaviours among adults across Europe [28].

Studies on beverage choice in different drinking cultures have primarily been conducted in the adult population, while research among adolescents is lacking. As beverage choice and related drinking behaviour vary substantially across age and sex [30,31], large differences in the distribution of the different beverage types consumed within drinking occasions, and changes over time, are to be expected between population subgroups such as adolescents, younger or older adults, or females and males. This means that the variations in beverage choice evident in previous characterisations of drinking cultures fail to capture differences in drinking patterns across various sub-populations [18,29]. This is particularly the case for adolescents, as general-population alcohol surveys do not usually include persons younger than 18 years of age [28]. However, accounting for recent developments in youth drinking, such as a decrease in alcohol consumption in this population, beverage choice and changes over time are likely to be different from adults in this group. In this study, we address this research gap by focusing specifically on adolescents’ drinking. Using an exploratory and data-driven approach, we (1) examined how the distribution of different alcoholic beverages (beer, wine, spirits and cider/alcopops) among girls and boys on recent drinking occasions varied over time and across European countries; (2) clustered adolescent beverage choice in European countries according to common beverage type distributions and trends; and (3) compared the resulting clusters with the prevalence of drinkers, and the prevalence of heavy drinkers and mean alcohol volume consumed on the last drinking day.

## 2. Methods

### 2.1. Data

Data on alcohol consumption were obtained from six waves (1999, 2003, 2007, 2011, 2015, and 2019) of the European School Survey Project on Alcohol and Other Drugs (ESPAD). In brief, cross-sectional school surveys were conducted every 4th year since 1995 for 15- to 16-year-old students. With the exception of Germany (Bavaria), in each participating country, sampling was carried out nationwide, using a stratified cluster sampling design. Data were collected in classrooms with either paper-and-pencil or online questionnaires. Participation was anonymous and voluntary. Different school types were included, with the class constituting the last unit of the multi-stage stratified sampling design [32]. For 24 countries, data on alcohol consumption were available for at least five waves; these countries were included in this study. The countries included were Bulgaria (BG), Croatia (HR), Cyprus (CY), the Czech Republic (CZ), Denmark (DK), Estonia (EE), Finland (FI), Germany (Bavaria) (DE), Greece (GR), Hungary (HU), Iceland (IS), Italy (IT), Latvia (LV), Lithuania (LT), Malta (MT), the Netherlands (NL), Norway (NO), Poland (PO), Portugal (PT), Romania (RO), the Slovak Republic (SK), Slovenia (SI), Sweden (SE) and Ukraine (UA).

### 2.2. Measures

The consumption of five different types of alcoholic beverage, beer, cider, alcopops, wine and spirits, was assessed for the last drinking day. To this end, participants were asked for each beverage separately, ‘If you drank beer/cider/alcopops/wine/spirits the last day you drank alcohol, how much did you drink?’. Beer/cider/alcopops were categorised as ‘<50 cl’, ‘50–100 cl’, ‘101–200 cl’ and ‘>200 cl’. The response categories for wine were ‘<10 cl’, ‘10–30 cl’, ’37 cl’, ‘≥75 cl’ until 2003, and changed to ‘<20 cl’, ‘20–40 cl’, ‘41–74 cl’ and ‘≥75 cl’ in 2007; the response categories for spirits were ‘<5 cl’, ‘5–10 cl’, ‘11–25 cl’ and ‘≥30 cl’ until 2003, and ‘<8 cl’, ‘8–15 cl’, ‘16–24 cl’ and ‘>25 cl’ from 2007. Quantities of pure alcohol per beverage type were converted into centilitres of pure alcohol using midpoints of the range of beverage quantities multiplied by the following alcohol concentrations: 5 vol% for beer, cider, and alcopops, 12 vol% for wine and 38 vol% for spirits [33]. For the present analysis, cider and alcopops were considered together. From this, the total amount of pure alcohol consumed on the last drinking day, and the distribution of four different beverage types (beer, wine, spirits, cider/alcopops) as the proportion of total alcohol intake reported as being consumed on the last drinking day, was calculated. To capture the prevalence of ‘heavy drinking’, a dichotomous variable was created with a cut-off set at five standard drinks of 12g of pure alcohol at the last drinking day. The prevalence of drinkers in the last 12 months was derived from a non-zero response to the question ‘On how many occasions (if any) have you had any alcoholic beverage to drink (…) during the past 12 months?’ A time variable with values ranging from 1 to 6 was generated for surveys conducted between 1999 and 2019 in four-year intervals.

### 2.3. Statistical Analyses

Due to the well-known sex differences in alcohol consumption, all analyses were conducted separately for girls and boys. Trends in the distribution of beverage types were analysed using fractional multinomial logit regression [34,35,36,37], a multivariate generalization of the fractional logit model proposed by Papke and Wooldridge [38]. Analyses were conducted using sample weights to control for the sample-specific characteristics of each country and using school as cluster variable for standard error adjustment (robust estimator of variance) [39,40,41]. The models were applied for each country, assuming the survey year to be a linear predictor. The analysis of linear trends was considered sufficient to capture the direction of change (increasing vs. decreasing). Further, the model requires a function of the same degree for all beverage types, i.e., no mixture of linear and quadratic or cubic trends in the beverage types. The proportions of total consumption represented by each beverage can vary between 0 and 100, adding up to 100% across all four beverage types for each participant. Consequently, the measures are mutually dependent and need to be treated as compositional data [42]. Cider/alcopops was used as a reference category. The resulting coefficients reflect the change in the proportion of beer, wine, and spirits, and must be interpreted relative to the change in the reference category. The predicted proportions of total consumption for each beverage (beer, wine, spirits, cider/alcopops) were calculated by country.

Based on the results from the fractional multinomial logit regression models for each country and sex, the trend coefficients for beer, wine and spirits, and the average predicted beverage proportions of total consumption (beer, wine, spirits, cider/alcopops) across all waves were used as variables in a hierarchical cluster analysis. The Ward’s method for linking and the Squared Euclidian Distance, an agglomerative approach, were applied on the basis of the variance of clusters. In each step, the sum of the squared distance of the individual cases from the respective cluster centroid was calculated for all pairs of clusters that were possibly being merged. The two clusters with the smallest increase in the total sum of squared distances were merged in the respective step [43,44].

Data from countries in the resulting clusters were pooled and the trend analyses, as described above, were applied within all clusters in girls and boys. Changes in beverage proportions across time were further examined by the difference between the highest and lowest rate between 2019 and 1999. The cut-off of 10 percentage points (pp) was used to consider the observed changes as diverging (positive difference ≥ 10 pp) or converging (negative difference ≤ 10 pp). Clusters were compared in terms of the prevalence of 12-month drinkers, mean alcohol volume and prevalence of heavy drinking on the last drinking day. Except for the prevalence of drinkers, the estimates were based on the subsample of the last 12-month drinkers. To account for different, country-specific sample sizes, these indicators were calculated for each cluster as the mean of the arithmetic country’s means across the whole observation period.

## 3. Results

Table A1 provides an overview of sample size by country and year. Table A2 shows the prevalence of drinkers and mean consumption of pure alcohol (cl) by country and year. The total sample size across all surveys and countries comprised 434,476 students; of these, 345,408 students reported any drinking in the last 12 months prior to the surveys.

### 3.1. Analysis of Beverage Choice with Multinomial Logit Regression

The results of the fractional multinominal regression analyses, as well as the model-predicted beverage choice by beverage, country and year, on which the following analyses are based, are depicted in Table A3, Table A4, Table A5 and Table A6. Across all countries, coefficients among girls and boys, respectively, ranged from −0.62 to 0.52 and −0.56 to 0.66 for beer, from −0.51 to 0.69 and −0.58 to 0.70 for wine, and from −0.95 to 0.91 and −0.70 to 0.95 for spirits. Regarding beverage choice, the predicted beverage proportions differed for girls and boys, respectively, and ranged from 7.5% to 78.5% and 31.1% to 84.0% for beer, 4.5% to 53.9% and 4.5% to 40.1% for wine, 2.2% to 43.0% and 1.6% to 24.7% for spirits and 1.6% to 61.8% and 1.4% to 46.3% for cider/alcopops.

### 3.2. Clusters of Country-Specific-Trends in Beverage Choice

**Figure 1:**
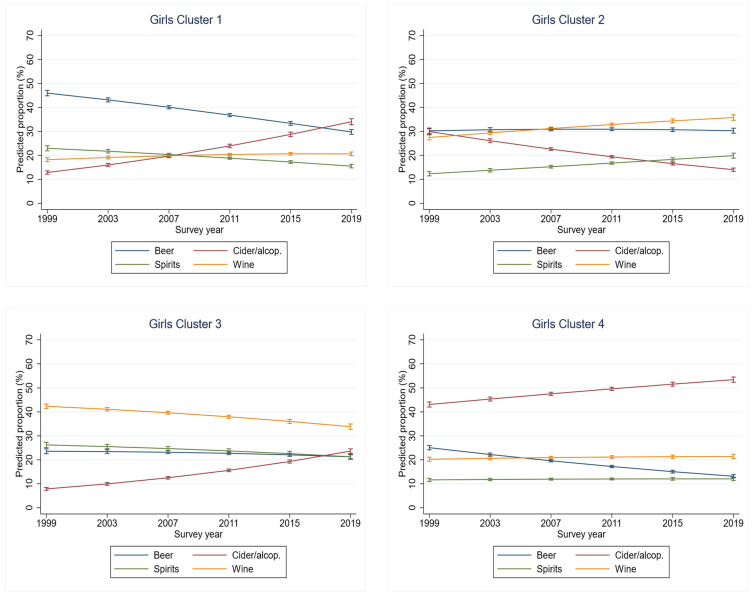
Clusters of trends in distribution of beverages consumed on most recent drinking occasions for girls.

**Figure 2:**
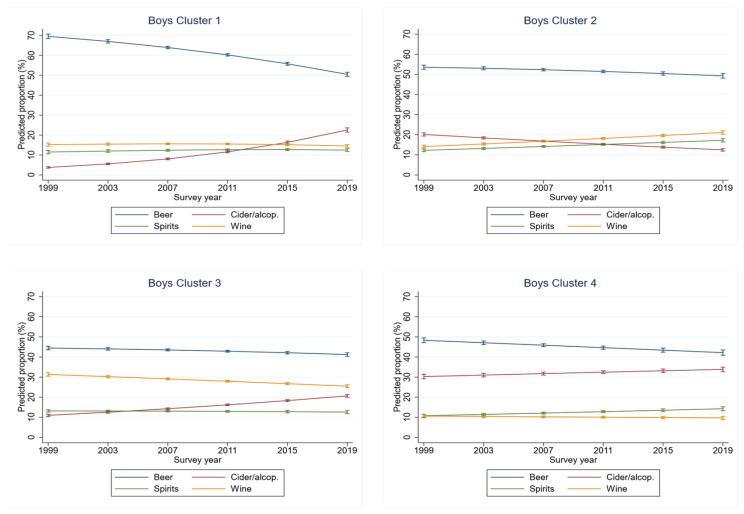
Clusters of trends in distribution of beverages consumed on most recent drinking occasions for boys.

The cluster analysis, which was conducted using the trend coefficients for each beverage type and the predicted proportions of beverage choice, yielded four distinct clusters for both girls and boys (Figure 1 and Figure 2). Among the girls (Figure 1), the first cluster, comprising Bulgaria, Cyprus, Iceland, Italy, Latvia, the Netherlands, Poland and Portugal, included the largest number of countries (Table 1). Over the twenty-year period, the proportion of cider/alcopops increased (+18 pp), while the proportion of beer declined (−15 pp) in this cluster. The second cluster, including Croatia, Greece, Lithuania, Romania and Germany, was characterised by a decrease in cider/alcopops over time (−14 pp). In the third cluster, comprising the Czech Republic, Hungary, Malta, Slovak Republic and Slovenia, beverage proportions converged over time. Here, the proportion of cider/alcopops increased over the twenty years (+18 pp), while the proportion of wine decreased (−7 pp). However, the wine proportion remained the highest proportion in this cluster. Finally, Denmark, Estonia, Finland, Norway, Sweden, and Ukraine constituted the fourth cluster, with a decreasing proportion of beer (−17 pp) and an increasing proportion of cider/alcopops (+21 pp) over time. The latter was the dominant beverage category across the observed period. This was the only case in which trends in beverage proportions were generally diverging.

Among boys (Figure 2), the first identified cluster included Bulgaria, Cyprus, Czech Republic, Latvia, the Netherlands, and Poland, with all countries other than Czech Republic also in the first cluster identified among girls (Table 2). In this cluster, the proportion of cider/alcopops increased (+16 pp) and the proportion of beer decreased (−19 pp) over time. Despite the decrease, beer remained the dominant beverage, with a mean share of 62% across all years. The second cluster, comprising Greece, Iceland, Lithuania, Portugal, Romania and Germany, was characterised by a high proportion of wine, second to beer, as the dominant beverage. The third cluster, including Croatia, Estonia, Hungary, Italy, Malta, Slovak Republic, Slovenia and Ukraine, was the cluster with the largest number of countries. Although the proportions of cider/alcopops increased over time (+12 pp), in this cluster, beer remained the dominant beverage. Finally, a fourth cluster, with Denmark, Finland, Norway and Sweden in common with the fourth cluster in girls, was identified. However, the characteristics of the clusters differed considerably between both sexes. Beer and cider/alcopops were the dominant beverages in boys. In girls, the proportion of cider/alcopops increased (16 pp) and the proportion of beer decreased (−11 pp); the levels of these beverages were also much lower and higher, respectively.

**Figure 3:**
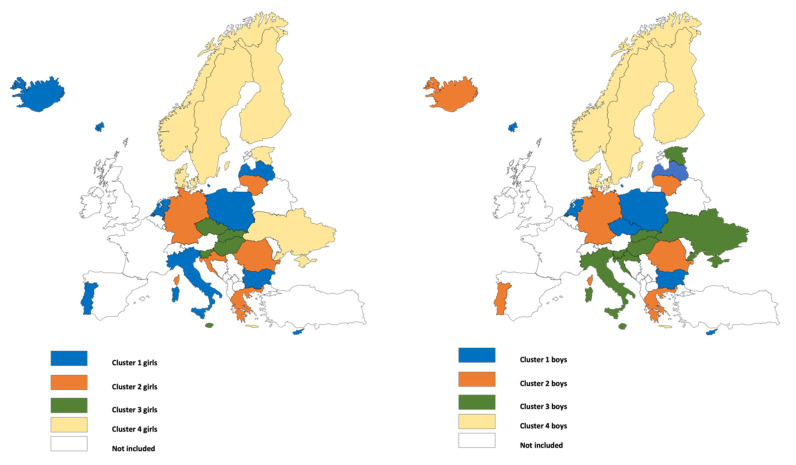
Geographical map of counties clustered by trends in beverage choice across the observation period (1999–2019) for girls (**left**) and boys (**right**).

Figure 3 graphically shows the classification of countries into clusters for girls and boys. Apart from the northern European countries (boys), including Estonia and Ukraine (girls), forming one cluster, no other cluster matched an approximate geographically defined region. Although this cluster comprised almost the same countries for both girls and boys, there are large sex differences with regard to beverage choice. In girls, cider and alcopops were the most preferred beverage over the 20-years period, while, for boys, beer was the beverage of choice. In general, the variability between clusters was larger for girls than boys. Various beverages emerged as dominant in the clusters for girls, while for boys, beer was the dominant beverage in all four clusters, with the proportion never falling below 40%. All other beverage types barely exceeded 25%; only wine (cluster 2) and cider/alcopops (cluster 4) temporarily exceeded this level (Figure 1 and Figure 2).

### 3.3. Differences in Indicators of Drinking between Clusters

Compared to the remaining clusters, the fourth cluster for both sexes, largely representing northern European countries, showed a higher overall mean alcohol volume and a higher prevalence of heavy drinking (Table 3 and Table 4). Conversely, the prevalence of drinkers was statistically significantly lower. Mean alcohol volume and the prevalence of heavy drinking in the remaining clusters were similar. However, in the first cluster of boys, both mean alcohol volume and the prevalence of heavy drinking were higher than in the second and third cluster, but lower than in the fourth cluster. Overall, the estimates for the two risky drinking indicators were considerably higher in boys than girls.

## 4. Discussion

We examined the beverage choices of 15–16-year-old adolescents on recent drinking occasions in 24 European countries and how they evolved over time. We investigated these changes separately for girls and boys and performed a cluster analysis to detect commonalities and differences between countries. Four clusters emerged for both sexes. Remarkably, in the majority of clusters, the preference for cider/alcopops increased over time, while the preference for more traditional beverages decreased. We found that risky drinking behaviours, in terms of high mean alcohol volume and high prevalence of heavy drinking on the most recent drinking day, were more common in northern European countries, Estonia, and Ukraine (in the latter two countries only among girls), the cluster where cider/alcopops were most (and, for boys, second most) preferred.

Previous research has found an association between a preference for spirits and heavy use in adolescents [5,9] and adults [28]. In contrast with the findings from Kilian and colleagues’ adult sample, spirits were not identified as the most preferred beverage in any cluster. In all clusters, the preference for spirits almost constantly ranked 3rd or 4th, indicating that, among 15–16-year-old adolescents in Europe, spirits are not typically preferred. Even though, as known from previous studies, drinking spirits is associated with a higher alcohol intake at the individual level, the present clusters may not differ in terms of indicators of risky drinking due to the generally low proportions of spirit consumption. This is not surprising, as the availability of spirits is limited for adolescents by youth protection legislation and for financial reasons.

Among girls in northern European countries (cluster 4), high and increasing proportions of cider/alcopops were associated with higher mean alcohol volume and a higher prevalence of heavy drinking on participants’ most recent drinking occasion. An increase in the proportion of cider and alcopops among the youngest cohort could also be found in a recent study on beverage preferences according to age, period and cohort in Sweden [30]. In contrast, a preference for beer—known to be associated with a higher alcohol intake and more alcohol-related problems [45,46,47]—was found to be comparatively low. The picture was different for boys: clusters with a high mean alcohol consumption and a high prevalence of heavy drinking, including the northern European countries (cluster 4), were characterised by a strong preference for beer, in combination with a high or increasing preference for cider/alcopops. Although the prevalence of monthly heavy drinking among adolescents has sharply declined in northern European countries, the level of heavy drinking is still comparable to those in other regions [48]. Despite these changes, adolescents in northern European countries seem to match the adult drinking habits that are traditionally described as a dry drinking culture [25,26]. Our findings on heavy drinking in northern European countries are similar to those reported by Bye and Rossow [49], who classified 13 European countries in terms of the incidence of intoxication. They found that Finland, Norway and Sweden formed the group with the highest rates of intoxication [49]. Apart from the northern European cluster for girls and boys, there was little overlap between our clusters and descriptions of geographically distinct drinking cultures in terms of beverage preference. For instance, no cluster showed the typically Mediterranean beverage preference for wine. Our findings highlight the importance of looking at young people separately.

Beverage choice among adolescents is a function of various aspects, including taste or predilection, as well as the availability of specific beverages. Accordingly, the distribution of the beverage types consumed on drinking occasions may reflect changes in availability. In Sweden, Norway and Finland, alcohol, with the exception of low-alcohol beverages, can only be purchased in government-run alcohol monopoly stores, where age limits are controlled more strictly than in grocery stores, in which beverages with a low alcohol content are sold [50]. Laws on alcopops have been eased in Norway over the observed time period; since 2003, they can be sold in grocery stores under the same conditions as low-alcohol beverages [51]. In Finland, the maximum alcohol content of beverages sold in grocery stores was raised from 4.7% to 5.5% in 2018. Such changes may partly underpin the evident increase in the preference for cider/alcopops over time, particularly in the northern European countries (cluster 4). From a public-health perspective, these developments may be problematic. We found increasing and, particularly in girls, high proportions of cider and alcopop consumption. This was most evident in cluster 4, which also showed the highest average prevalence of heavy drinking and mean consumption. These are likely to lead to corresponding increases in harm. These results have implications for prevention strategies and policies aimed at reducing alcohol-related harms, such as increasing alcohol excise duties to reduce the affordability of alcoholic beverages. For example, the work of Meier and colleagues [52] suggests that these interventions have differential effects according to gender, age, drinking level and context; increasing the price of cheap on-trade or on-trade and off-trade alcohol affects young, male heavy drinkers. Similarly, beverage-specific taxes such as the so-called ‘alcopops’ taxes have been shown to reduce the consumption of alcopops [53]. However, the taxation of alcopops should be part of a holistic alcohol taxation strategy in which all alcoholic beverages are taxed according to their alcohol content, so that a reduction in overall consumption is a priority, and major shifts towards cheaper products are avoided. Reducing the alcohol content of the respective drinks, so that less alcohol is consumed with the same fluid intake [54], or increasing the minimum age of consumption from 16 to 18 years could also be effective [55]. Importantly, these beverage-specific, tailored and targeted strategies can have wider benefits across consumption, including broader alcohol consumption levels and links to related outcomes, such as sexually transmitted infections [56] and other harms [57].

Most importantly, recent discussions on drinking cultures have already extended beyond the subject of traditionally preferred beverages [18,29,58]. When describing and distinguishing drinking cultures in adults as well as adolescents, several aspects must be considered, with beverage preference being only one of them. These include, but are not limited to, the proportion of abstainers, the prevalence of heavy drinking, average alcohol intake per drinking day and the drinking context [18,28]. In terms of beverage preference, our results indicate that adolescents’ choices do not mirror population-level approaches to drinking culture or classifications by dominant beverages. The traditional geographical division between countries does not neatly apply to our findings with adolescents. Additionally, viewing drinking cultures as homogeneous units is not satisfactory. While the distinction between dry/wet drinking cultures considers supranational similarities, it neglects within-country differences [29]. This implies that the traditional concepts of drinking cultures may not apply to adolescents, and suggests a need to broaden the understanding of drinking culture and develop new concepts and theories.

A generic view of drinking cultures tends to result in stereotypes that do not reflect the variation in actual consumption behaviour. The notion of there being ‘many drinking cultures’ within a society has recently come to the fore. Kilian and colleagues postulated the existence of several combinations of drinking cultures within a country rather than only one [28]. Descriptions below the macro-level that consider sub-societal entities already exist. However, they primarily refer to ethnic subgroups [29,59,60], sex e.g., [8,61], social class [62] or subcultures, such as lifestyle or music taste [29,63,64], rather than adolescents. The findings of Kraus and colleagues [30] point towards strong age effects regarding beverage choice. We would thus argue that adolescents represent a subgroup whose drinking behaviour is subject to specific influences from their peer group, social media, or celebrities, which need to be considered separately. In addition, there are changes related to external influences that are independent of age, such as technical developments, the economic situation or globalisation [65]. These may affect some groups more than others, and may particularly affect adolescents, as they are in their formative years [66,67]. This might eventually lead to the observed variations in adults’ and adolescents’ beverage preferences.

### Limitations

ESPAD data on alcohol consumption derive from self-reported consumption on the last drinking day. Hence, the reported amount and type of alcohol consumed may not be representative of an individual’s drinking behaviour [68,69] and may be underreported [70,71]. However, trends in beverage choice will be unaffected, as long as underreporting by beverage type does not change considerably over time [72]. Second, hierarchical cluster analysis lacks statistical decision criteria, leading to an arbitrary number of clusters. However, comparisons with solutions with more or fewer clusters did not result in geographically distinct clusters or clusters with a high mean alcohol volume that did not contain northern European countries, Estonia and Ukraine (data available on request). Third, in 2007, some response categories regarding quantities of wine and spirits consumption were slightly changed, with shifts to higher and lower quantities, respectively. However, no systematic biases are expected to affect the main results because individual beverage proportions are calculated based on total alcohol volume, which is modified by the changed categories to the same extent as the volume of the specific beverage (i.e., wine, spirits) that was consumed. Thus, we expect only minor shifts in the beverage proportions. Moreover, as these changes equally apply to all countries, between-country comparisons are not affected. Fourth, the weight of individual countries in the clusters varies according to sample size. As the fractional multinomial logit regression model for calculating within-cluster trends contains country-specific weights to control for sample characteristics and adjust for the standard error individuals nested within schools, additional weighting by sample size was not possible. Similarly, using the mean of the arithmetic country means that the regression analysis of each cluster results in model overspecification. However, sensitivity analyses based on the raw data revealed only marginal differences, justifying the use of pooled data. Finally, clusters of trends in beverage preference were compared with indicators of drinking (prevalence of drinking, mean alcohol volume and prevalence of heavy drinking) across the entire observation period. Hence, the associations between changes in beverage preferences and average consumption indicators need to be cautiously interpreted.

## 5. Conclusions

Temporal trends in beverage preferences among adolescents vary considerably between European countries and between girls and boys. Adolescent beverage choices and changes in beverage choice are associated with alcohol volume and heavy drinking, particularly with the high and increasing proportions of the use of cider/alcopops. This points to the necessity of considering beverage choice in alcohol policies. Measures such as the adaptation of taxes, and minimum pricing policies for beverages associated with risky drinking behaviour, might be effective here. In addition to clusters consisting of primarily northern European countries, the resulting clusters do not match the previous findings of geographically distinct regions, representing particular drinking cultures. The present results support the recent debate on drinking cultures, suggesting the importance of beverage choice and considering various drinking patterns within countries. Future approaches to the study of drinking cultures should broaden their scope by acknowledging adolescents as a distinct subpopulation.

## Figures and Tables

**Table 1:** Clusters by country, dominant beverage(s), trends, and difference between highest and lowest in 2019 and highest and lowest in 1999 for girls.

Cluster	Countries	Dominant Beverage(s) (over Years)	Trends ^1^	Difference (Highest–Lowest 2019 Beverage Minus Highest–Lowest Beverage in 1999)
1	Bulgaria, Cyprus, Iceland, Italy, Latvia, Netherlands, Poland, and Portugal	Beer (40%) Cider/alcopops emerging (20%)	Beer ↓ Cider/alcopops ↑	−26.06 pp; converging
2	Croatia, Greece, Lithuania, Romania, Germany	Wine (32%) Beer (31%) Spirits (16%)	Cider/alcopops ↓	3.18 pp; stable
3	Czech Republic, Hungary, Malta, Slovak Republic, and Slovenia	Wine (39%) Spirits (24%)	Wine ↓ Cider/alcopops ↑	−41.53 pp; converging
4	Denmark, Estonia, Finland, Norway, Sweden, and Ukraine	Cider/alcopops (47%)	Cider/alcopops ↑ Beer ↓	24.48 pp; diverging

^1^ Presentation of a selection of trends that particularly shape the development in the respective clusters; ↓ decreasing trend; ↑ increasing trend.

**Table 2:** Clusters by country, dominant beverage(s), trends, and difference between highest–lowest 2019 and highest–lowest in 1999 for boys.

Cluster	Countries	Dominant Beverage(s) (over Years)	Trends	Difference (Highest–Lowest 2019 Beverage Minus Highest–Lowest Beverage in 1999)
1	Bulgaria, Cyprus, Czech Republic, Latvia, Netherlands, Poland	Beer (62%) Cider/alcopops (11%)	Beer ↓ Cider/alcopops ↑	−22.63 pp; converging
2	Greece, Iceland, Lithuania, Portugal, Romania, Germany	Beer (43%) Wine (29%)	Cider/alcopops ↑	−9.52 pp; converging
3	Croatia, Estonia, Hungary, Italy, Malta, Slovak Republic, Slovenia Ukraine	Beer (52%)	Cider/alcopops ↓	−3.64 pp; stable
4	Denmark, Finland, Norway, and Sweden	Beer (47%) Cider/alcopops (30%)	Beer ↓	−8.17 pp; converging

↓ decreasing trend; ↑ increasing trend.

**Table 3:** Clusters by country and indicators of drinking for girls.

Cluster	Country	Prevalence of Drinkers (%, SD)	Heavy Drinking ^1^ (%, SD)	Alcohol Volume ^1^ (Mean, SD)
1	Bulgaria	71.96 (7.41)	14.08 (8.08)	3.47 (0.97)
	Cyprus			
	Iceland			
	Italy			
	Latvia			
	Netherlands			
	Poland			
	Portugal			
2	Croatia	84.27 (5.76)	11.92 (5.62)	3.37 (0.79)
	Greece			
	Lithuania			
	Romania			
	Germany			
3	Czech Rep.	86.85 (2.95)	10.66 (2.91)	3.12 (0.41)
	Hungary			
	Malta			
	Slovak Rep.			
	Slovenia			
4	Denmark	78.93 (7.33)	26.37 (9.45)	5.03 (0.96)
	Estonia			
	Finland			
	Norway			
	Sweden			
	Ukraine			

^1^ Drinkers only.

**Table 4:** Clusters by country and indicators of drinking for boys.

Cluster	Country	Prevalence of Drinkers (%, SD ^1^)	Heavy Drinking ^1^ (%, SD)	Alcohol Volume ^1^ (Mean, SD)
1	Bulgaria	81.27 (7.09)	30.54 (7.64)	5.36 (0.54)
	Cyprus			
	Czech Rep.			
	Latvia			
	Netherlands			
	Poland			
2	Greece	78.63 (12.23)	25.04 (8.48)	4.85 (0.94)
	Iceland			
	Lithuania			
	Portugal			
	Romania			
	Germany			
3	Croatia	85.92 (2.06)	24.91 (5.41)	5.11 (0.69)
	Estonia			
	Hungary			
	Italy			
	Malta			
	Slovak Rep.			
	Slovenia			
	Ukraine			
4	Denmark	76.47 (10.07)	43.89 (6.92)	6.75 (0.84)
	Finland			
	Norway			
	Sweden			

^1^ Drinkers only.

## Data Availability

The ESPAD trend data are archived at the Italian National Research Council (CNR) where data can be applied for research purposes.

## Appendix A

**Table A1:** Sample size, by country and year (1999–2019), including abstainers.

Country	1999	2003	2007	2011	2015	2019
Bulgaria	–	2666	2353	2217	2922	2864
Croatia	3555	2852	3008	3002	2558	2772
Cyprus	–	2142	6340	4243	2098	1224
Czech Republic	3543	3149	3901	3913	2738	2778
Denmark	1546	2504	877	2181	1670	2488
Estonia	–	2431	2372	2460	2452	2520
Finland	3003	3219	4988	3744	4049	4594
Germany	–	4219	5011	2796	862	1459
Greece	2195	1891	3060	5908	3202	5988
Hungary	2726	3109	2817	3063	2735	2423
Iceland	3457	3313	3510	3333	2663	2534
Italy	4073	4818	9981	4837	4059	2542
Latvia	2296	2816	–	2622	1119	2743
Lithuania	–	5028	2411	2476	2573	2393
Malta	3635	3443	3668	3377	3326	3043
Netherlands	2613	2068	2091	2044	1684	1288
Norway	3753	3745	3482	2938	2584	4313
Poland	3269	5842	2120	5934	11,822	5047
Portugal	3577	2919	3141	1965	3456	4365
Romania	2368	4323	2289	2770	3500	3764
Slovak Republic	2437	2122	2468	2009	2208	2258
Slovenia	2347	2758	3085	3186	3484	3413
Sweden	3271	3212	3179	2569	2551	2546
Ukraine	2958	4114	2447	2210	2350	2731

**Table A2:** Prevalence of drinkers and mean consumption (drinkers only) in pure alcohol (cl) by country and year (1999–2019).

Country	Prevalence of Drinkers						Mean Consumption					
	1999	2003	2007	2011	2015	2019	1999	2003	2007	2011	2015	2019
Bulgaria	–	80%	84%	87%	86%	81%	–	3.49	4.20	3.88	4.22	3.66
Croatia	80%	88%	86%	88%	85%	81%	4.28	5.31	4.97	5.41	4.61	4.93
Cyprus	–	85%	65%	85%	84%	78%	–	5.23	3.16	4.30	4.28	3.67
Czech Republic	91%	94%	94%	93%	93%	91%	4.74	5.07	5.14	5.00	4.74	4.72
Denmark	95%	94%	92%	92%	90%	88%	6.45	7.67	6.89	7.90	7.94	6.38
Estonia	–	92%	92%	93%	85%	80%	6.46	5.47	5.87	4.50	4.11	6.46
Finland	90%	84%	81%	80%	69%	67%	7.07	6.32	6.02	6.09	5.05	4.73
Germany	–	91%	92%	81%	79%	83%	–	6.96	4.74	4.68	4.65	4.32
Greece	92%	93%	88%	90%	91%	86%	3.07	4.34	3.10	3.71	2.98	3.09
Hungary	76%	84%	87%	91%	89%	88%	2.47	3.62	3.68	4.19	4.04	4.25
Iceland	78%	72%	63%	49%	30%	31%	6.08	6.57	5.72	3.57	3.47	3.43
Italy	80%	81%	85%	82%	81%	80%	2.75	3.38	4.09	3.95	4.16	4.35
Latvia	82%	86%	–	89%	78%	82%	2.90	4.48	–	4.61	4.21	3.62
Lithuania	–	96%	91%	93%	81%	69%	–	5.74	4.35	4.44	3.28	2.33
Malta	91%	89%	88%	86%	82%	77%	4.50	5.00	3.39	3.35	2.91	3.07
Netherlands	75%	66%	88%	82%	67%	68%	5.11	5.90	5.17	5.52	5.38	6.07
Norway	82%	80%	74%	66%	55%	49%	5.25	7.41	7.06	6.20	5.19	4.59
Poland	80%	81%	81%	81%	76%	75%	4.73	5.18	4.19	4.65	4.24	4.11
Portugal	71%	63%	73%	80%	67%	62%	3.22	3.09	2.10	2.52	2.44	2.86
Romania	82%	87%	79%	75%	67%	73%	2.98	4.59	3.41	3.29	2.81	2.77
Slovak Republic	87%	88%	89%	87%	86%	87%	3.01	3.55	3.67	3.87	3.87	4.11
Slovenia	85%	86%	91%	89%	84%	79%	4.35	4.82	4.99	4.77	3.92	3.78
Sweden	84%	79%	75%	68%	57%	51%	6.05	5.80	5.57	5.67	4.65	3.34
Ukraine	85%	84%	82%	86%	70%	82%	3.46	3.52	3.89	3.70	3.43	5.10

**Table A3:** Regression coefficients of time trends in beverage proportions with reference category cider/alcopops for girls.

Country		Coefficient	SE	Country		Coefficient	SE
Bulgaria	spirits	−0.191 ***	−0.047	Latvia	spirits	−0.245 ***	−0.027
	beer	−0.511 ***	−0.037		beer	−0.357 ***	−0.021
	wine	0.04	−0.045		wine	−0.338 ***	−0.024
Croatia	spirits	0.247 ***	−0.024	Lithuania	spirits	0.493 ***	−0.038
	beer	0.121 ***	−0.025		beer	0.061 **	−0.030
	wine	0.200 ***	−0.024		wine	0.290 ***	−0.024
Cyprus	spirits	−0.497 ***	−0.067	Malta	spirits	−0.280 ***	−0.028
	beer	−0.584 ***	−0.084		beer	−0.332 ***	−0.026
	wine	−0.482 ***	−0.070		wine	−0.334 ***	−0.023
Czech Rep.	spirits	−0.545 ***	−0.030	Netherlands	spirits	−0.947 ***	−0.057
	beer	−0.523 ***	−0.026		beer	−0.623 ***	−0.042
	wine	−0.513 ***	−0.030		wine	−0.241***	−0.029
Denmark	spirits	0.122 *	−0.065	Norway	spirits	0.151 ***	−0.033
	beer	−0.201 **	−0.081		beer	−0.091 ***	−0.027
	wine	0.212 ***	−0.078		wine	−0.080 **	−0.034
Estonia	spirits	0.029	−0.029	Poland	spirits	−0.180 ***	−0.040
	beer	−0.159 ***	−0.032		beer	−0.301 ***	−0.038
	wine	0.052 **	−0.024		wine	−0.268 ***	−0.039
Finland	spirits	0.035	−0.024	Portugal	spirits	−0.100 ***	−0.019
	beer	−0.127 ***	−0.017		beer	−0.124 ***	−0.024
	wine	0.0315	−0.021		wine	0.085 ***	−0.028
Germany	spirits	0.029	−0.035	Romania	spirits	0.912 ***	−0.049
	beer	0.134 ***	−0.028		beer	0.522 ***	−0.037
	wine	0.000	−0.032		wine	0.692 ***	−0.039
Greece	spirits	−0.014	−0.025	Slovak Republic	spirits	−0.391 ***	−0.028
	beer	0.049 *	−0.027		beer	−0.341 ***	−0.030
	wine	0.095 ***	−0.023		wine	−0.467 ***	−0.028
Hungary	spirits	−0.319 ***	−0.031	Slovenia	spirits	0.040	−0.023
	beer	−0.081 ***	−0.025		beer	−0.027	−0.023
	wine	−0.100 ***	−0.023		wine	−0.089 ***	−0.023
Iceland	spirits	−0.107 ***	−0.034	Sweden	spirits	−0.018	−0.027
	beer	−0.284 ***	−0.02		beer	−0.327 ***	−0.021
	wine	−0.086 **	−0.037		wine	−0.268 ***	−0.025
Italy	spirits	−0.701 ***	−0.033	Ukraine	spirits	−0.413 ***	−0.038
	beer	−0.359 ***	−0.024		beer	−0.059 ***	−0.018
	wine	−0.383 ***	−0.028		wine	0.043 **	−0.017

* *p* < 0.1; ** *p* < 0.05; *** *p* < 0.001.

**Table A4:** Regression coefficients of time trends in beverage proportions with reference category cider/alcopops for boys.

Country		Coefficient	SE	Country		Coefficient	SE
Bulgaria	spirits	−0.122 **	−0.054	Latvia	spirits	−0.159 ***	−0.033
	beer	−0.498 ***	−0.035		beer	−0.315 ***	−0.023
	wine	−0.241 ***	−0.043		wine	−0.277 ***	−0.029
Croatia	spirits	0.072 **	−0.028	Lithuania	spirits	0.471 ***	−0.036
	beer	−0.017	−0.023		beer	0.124 ***	−0.024
	wine	0.011	−0.024		wine	0.250 ***	−0.029
Cyprus	spirits	−0.587 ***	−0.055	Malta	spirits	−0.123 ***	−0.026
	beer	−0.545 ***	−0.045		beer	−0.250 ***	−0.021
	wine	−0.577 ***	−0.053		wine	−0.267 ***	−0.025
Czech Rep.	spirits	−0.435 ***	−0.031	Netherlands	spirits	−0.703 ***	−0.057
	beer	−0.555 ***	−0.027		beer	−0.440 ***	−0.028
	wine	−0.489 ***	−0.032		wine	−0.381 ***	−0.052
Denmark	spirits	0.231 ***	−0.058	Norway	spirits	0.223 ***	−0.032
	beer	0.031	−0.044		beer	0.105 ***	−0.024
	wine	0.190 ***	−0.068		wine	0.020	−0.037
Estonia	spirits	0.018	−0.029	Poland	spirits	−0.188 ***	−0.043
	beer	−0.156 ***	−0.022		beer	−0.300 ***	−0.04
	wine	0.003	−0.025		wine	−0.285 ***	−0.041
Finland	spirits	0.025	−0.028	Portugal	spirits	−0.034 *	−0.02
	beer	0.004	−0.016		beer	−0.069 ***	−0.019
	wine	0.026	−0.025		wine	0.074 ***	−0.027
Germany	spirits	0.059	−0.045	Romania	spirits	0.952 ***	−0.051
	beer	0.096 ***	−0.035		beer	0.658 ***	−0.039
	wine	−0.038	−0.046		wine	0.701 ***	−0.041
Greece	spirits	0.049 *	−0.026	Slovak Republic	spirits	−0.376 ***	−0.039
	beer	0.057 **	−0.026		beer	−0.405 ***	−0.035
	wine	0.010	−0.023		wine	−0.540 ***	−0.039
Hungary	spirits	−0.222 ***	−0.032	Slovenia	spirits	0.140 ***	−0.029
	beer	−0.116 ***	−0.024		beer	−0.011	−0.018
	wine	−0.205 ***	−0.024		wine	−0.086 ***	−0.02
Iceland	spirits	0.011	−0.031	Sweden	spirits	−0.012	−0.024
	beer	−0.097 ***	−0.023		beer	−0.186 ***	−0.017
	wine	0.052	−0.034		wine	−0.170 ***	−0.031
Italy	spirits	−0.507 ***	−0.029	Ukraine	spirits	−0.434 ***	−0.035
	beer	−0.285 ***	−0.021		beer	−0.103 ***	−0.022
	wine	−0.334 ***	−0.025		wine	−0.008	−0.022

* *p* < 0.1; ** *p* < 0.05; *** *p* < 0.001.

**Table A5:** Model predictions of beverage proportions for girls, 1999–2019 (margins, SE).

Country	Year						
		1999	2003	2007	2011	2015	2019
BG	spirits	0.101	0.125	0.147	0.164	0.173	0.172
		−0.015	−0.013	−0.010	−0.007	−0.006	−0.010
	wine	0.0549	0.0853	0.127	0.178	0.237	0.297
		−0.008	−0.009	−0.009	−0.007	−0.007	−0.012
	beer	0.785	0.702	0.600	0.487	0.372	0.269
		−0.020	−0.018	−0.014	−0.009	−0.008	−0.011
	cider/alcop.	0.0594	0.0885	0.126	0.171	0.218	0.262
		−0.008	−0.008	−0.008	−0.007	−0.007	−0.011
HR	spirits	0.156	0.173	0.190	0.208	0.226	0.245
		−0.007	−0.006	−0.006	−0.006	−0.008	−0.011
	wine	0.330	0.348	0.366	0.382	0.396	0.408
		−0.010	−0.009	−0.008	−0.009	−0.010	−0.012
	beer	0.297	0.291	0.282	0.272	0.261	0.248
		−0.010	−0.007	−0.005	−0.005	−0.007	−0.009
	cider/alcop.	0.217	0.188	0.162	0.138	0.117	0.0991
		−0.009	−0.006	−0.005	−0.006	−0.006	−0.007
CY	spirits	0.197	0.195	0.187	0.170	0.147	0.118
		−0.028	−0.019	−0.011	−0.007	−0.009	−0.012
	wine	0.232	0.233	0.226	0.210	0.183	0.149
		−0.029	−0.020	−0.013	−0.009	−0.010	−0.014
	beer	0.495	0.449	0.394	0.330	0.260	0.191
		−0.039	−0.026	−0.016	−0.015	−0.022	−0.028
	cider/alcop.	0.0756	0.123	0.193	0.290	0.410	0.542
		−0.016	−0.018	−0.019	−0.021	−0.029	−0.040
CZ	spirits	0.213	0.204	0.193	0.179	0.161	0.138
		−0.008	−0.006	−0.004	−0.004	−0.005	−0.006
	wine	0.385	0.381	0.372	0.356	0.330	0.293
		−0.010	−0.007	−0.006	−0.006	−0.008	−0.011
	beer	0.372	0.365	0.353	0.335	0.307	0.270
		−0.010	−0.007	−0.005	−0.006	−0.007	−0.008
	cider/alcop.	0.030	0.050	0.082	0.130	0.202	0.299
		−0.003	−0.004	−0.004	−0.005	−0.006	−0.011
DK	spirits	0.066	0.077	0.089	0.102	0.115	0.128
		−0.015	−0.013	−0.010	−0.006	−0.006	−0.012
	wine	0.045	0.058	0.074	0.093	0.114	0.139
		−0.011	−0.011	−0.010	−0.007	−0.007	−0.013
	beer	0.317	0.270	0.227	0.188	0.153	0.124
		−0.055	−0.035	−0.019	−0.009	−0.011	−0.016
	cider/alcop.	0.572	0.595	0.610	0.618	0.617	0.609
		−0.057	−0.039	−0.025	−0.015	−0.015	−0.022
EE	spirits	0.108	0.111	0.115	0.117	0.120	0.122
		−0.009	−0.006	−0.005	−0.004	−0.005	−0.007
	wine	0.265	0.282	0.298	0.313	0.328	0.343
		−0.014	−0.010	−0.008	−0.007	−0.009	−0.013
	beer	0.166	0.142	0.122	0.104	0.088	0.075
		−0.013	−0.008	−0.005	−0.004	−0.004	−0.005
	cider/alcop.	0.461	0.465	0.466	0.466	0.464	0.460
		−0.016	−0.012	−0.008	−0.007	−0.009	−0.013
FI	spirits	0.093	0.099	0.104	0.110	0.115	0.121
		−0.005	−0.004	−0.003	−0.004	−0.005	−0.008
	wine	0.137	0.145	0.153	0.160	0.167	0.175
		−0.007	−0.005	−0.004	−0.004	−0.006	−0.009
	beer	0.258	0.233	0.209	0.187	0.167	0.149
		−0.009	−0.006	−0.005	−0.004	−0.005	−0.007
	cider/alcop.	0.511	0.523	0.534	0.543	0.550	0.556
		−0.010	−0.007	−0.005	−0.005	−0.008	−0.011
DE	spirits	0.102	0.101	0.101	0.099	0.098	0.096
		−0.007	−0.005	−0.004	−0.004	−0.006	−0.008
	wine	0.383	0.370	0.356	0.342	0.327	0.312
		−0.014	−0.009	−0.006	−0.008	−0.013	−0.017
	beer	0.222	0.245	0.270	0.296	0.324	0.353
		−0.010	−0.008	−0.007	−0.008	−0.011	−−0.015
	cider/alcop.	0.294	0.284	0.274	0.263	0.251	0.239
		−0.014	−0.009	−0.006	−0.007	−0.011	−0.014
GR	spirits	0.296	0.283	0.270	0.257	0.245	0.232
		−0.012	−0.008	−0.006	−0.006	−0.008	−0.010
	wine	0.219	0.233	0.248	0.264	0.280	0.296
		−0.008	−0.007	−0.005	−0.005	−0.007	−0.010
	beer	0.280	0.285	0.290	0.294	0.297	0.300
		−0.012	−0.009	−0.006	−0.006	−0.007	−0.011
	cider/alcop.	0.204	0.198	0.192	0.185	0.178	0.172
		−0.013	−0.010	−0.007	−0.006	−0.006	−0.007
HU	spirits	0.368	0.313	0.263	0.218	0.178	0.144
		−0.013	−0.009	−0.006	−0.006	−0.007	−0.008
	wine	0.357	0.379	0.396	0.408	0.416	0.419
		−0.010	−0.007	−0.006	−0.006	−0.008	−0.011
	beer	0.147	0.158	0.169	0.177	0.184	0.189
		−0.007	−0.006	−0.004	−0.004	−0.006	−0.008
	cider/alcop.	0.128	0.150	0.173	0.197	0.222	0.247
		−0.009	−0.007	−0.006	−0.006	−0.007	−0.011
IS	spirits	0.150	0.159	0.167	0.172	0.174	0.174
		−0.010	−0.007	−0.006	−0.008	−0.011	−0.014
	wine	0.0897	0.0973	0.104	0.109	0.113	0.116
		−0.007	−0.005	−0.004	−0.005	−0.008	−0.011
	beer	0.531	0.472	0.414	0.357	0.304	0.254
		−0.011	−0.008	−0.006	−0.008	−0.010	−0.012
	cider/alcop.	0.229	0.271	0.316	0.362	0.409	0.455
		−0.009	−0.008	−0.008	−0.010	−0.014	−0.019
IT	spirits	0.353	0.270	0.197	0.137	0.091	0.058
		−0.016	−0.009	−0.005	−0.004	−0.005	−0.005
	wine	0.211	0.221	0.222	0.213	0.194	0.168
		−0.011	−0.007	−0.005	−0.005	−0.007	−0.009
	beer	0.351	0.378	0.389	0.381	0.355	0.316
		−0.014	−0.009	−0.006	−0.006	−0.008	−0.011
	cider/alcop.	0.085	0.130	0.192	0.269	0.360	0.458
		−0.005	−0.006	−0.005	−0.005	−0.009	−0.014
LV	spirits	0.126	0.130	0.132	0.130	0.126	0.119
		−0.008	−0.007	−0.005	−0.005	−0.006	−0.008
	wine	0.395	0.371	0.342	0.308	0.272	0.233
		−0.014	−0.010	−0.008	−0.009	−0.010	−0.013
	beer	0.331	0.305	0.275	0.244	0.210	0.177
		−0.013	−0.009	−0.007	−0.007	−0.008	−0.009
	cider/alcop.	0.148	0.194	0.251	0.318	0.392	0.471
		−0.008	−0.008	−0.007	−0.008	−0.010	−0.013
LT	spirits	0.0372	0.054	0.077	0.107	0.144	0.189
		−0.004	−0.004	−0.004	−0.004	−0.007	−0.013
	wine	0.254	0.301	0.350	0.396	0.435	0.465
		−0.011	−0.009	−0.007	−0.008	−0.011	−0.016
	beer	0.267	0.252	0.233	0.210	0.183	0.156
		−0.013	−0.008	−0.005	−0.006	−0.007	−0.009
	cider/alcop.	0.441	0.392	0.340	0.288	0.237	0.190
		−0.015	−0.011	−0.008	−0.008	−0.010	−0.011
MT	spirits	0.331	0.335	0.335	0.332	0.324	0.312
		−0.009	−0.007	−0.007	−0.008	−0.011	−0.014
	wine	0.418	0.400	0.379	0.355	0.329	0.299
		−0.009	−0.007	−0.006	−0.007	−0.009	−0.011
	beer	0.183	0.175	0.166	0.156	0.145	0.132
		−0.006	−0.005	−0.004	−0.005	−0.006	−0.007
	cider/alcop.	0.0676	0.0902	0.119	0.156	0.202	0.257
		−0.005	−0.005	−0.004	−0.004	−0.006	−0.011
NL	spirits	0.412	0.289	0.180	0.100	0.0505	0.0235
		−0.022	−0.012	−0.008	−0.008	−0.006	−0.004
	wine	0.143	0.204	0.258	0.290	0.296	0.279
		−0.010	−0.009	−0.008	−0.008	−0.010	−0.013
	beer	0.353	0.342	0.295	0.227	0.158	0.102
		−0.019	−0.011	−0.008	−0.009	−0.011	−0.011
	cider/alcop.	0.0918	0.166	0.267	0.383	0.496	0.596
		−0.008	−0.009	−0.010	−0.013	−0.017	−0.021
NO	spirits	0.0883	0.105	0.123	0.145	0.169	0.195
		−0.009	−0.008	−0.007	−0.007	−0.010	−0.014
	wine	0.141	0.132	0.124	0.115	0.107	0.098
		−0.012	−0.008	−0.006	−0.006	−0.007	−0.009
	beer	0.252	0.234	0.217	0.200	0.183	0.166
		−0.016	−0.012	−0.009	−0.008	−0.009	−0.011
	cider/alcop.	0.519	0.529	0.536	0.540	0.542	0.540
		−0.016	−0.012	−0.010	−0.010	−0.013	−0.017
PO	spirits	0.097	0.106	0.117	0.127	0.139	0.150
		−0.006	−0.005	−0.004	−0.004	−0.005	−0.007
	wine	0.214	0.215	0.217	0.217	0.216	0.215
		−0.008	−0.006	−0.005	−0.005	−0.006	−0.007
	beer	0.674	0.657	0.639	0.619	0.597	0.573
		−0.009	−0.007	−0.006	−0.006	−0.007	−0.010
	cider/alcop.	0.016	0.021	0.028	0.037	0.048	0.062
		−0.002	−0.002	−0.002	−0.002	−0.003	−0.005
PT	spirits	0.430	0.421	0.409	0.397	0.382	0.367
		−0.011	−0.008	−0.006	−0.006	−0.007	−0.010
	wine	0.0737	0.0866	0.101	0.118	0.137	0.158
		−0.006	−0.005	−0.005	−0.004	−0.005	−0.008
	beer	0.340	0.325	0.309	0.292	0.275	0.257
		−0.013	−0.009	−0.007	−0.006	−0.007	−0.009
	cider/alcop.	0.156	0.168	0.181	0.193	0.206	0.218
		−0.008	−0.007	−0.005	−0.005	−0.006	−0.009
RO	spirits	0.0225	0.0382	0.0600	0.0883	0.124	0.166
		−0.003	−0.004	−0.004	−0.004	−0.005	−0.008
	wine	0.150	0.204	0.257	0.304	0.341	0.368
		−0.008	−0.007	−0.006	−0.007	−0.008	−0.011
	beer	0.413	0.474	0.504	0.503	0.476	0.434
		−0.014	−0.009	−0.007	−0.007	−0.009	−0.011
	cider/alcop.	0.415	0.283	0.178	0.106	0.0593	0.0320
		−0.017	−0.009	−0.005	−0.006	−0.005	−0.004
SK	spirits	0.229	0.233	0.237	0.238	0.237	0.232
		−0.012	−0.010	−0.008	−0.007	−0.008	−0.010
	wine	0.539	0.510	0.479	0.447	0.412	0.374
		−0.012	−0.010	−0.008	−0.008	−0.010	−0.012
	beer	0.217	0.233	0.249	0.263	0.275	0.283
		−0.009	−0.007	−0.006	−0.006	−0.009	−0.012
	cider/alcop.	0.016	0.023	0.035	0.052	0.077	0.111
		−0.002	−0.002	−0.002	−0.003	−0.004	−0.006
SI	spirits	0.171	0.184	0.198	0.212	0.227	0.243
		−0.009	−0.008	−0.007	−0.006	−0.008	−0.010
	wine	0.432	0.410	0.388	0.366	0.344	0.323
		−0.011	−0.008	−0.007	−0.008	−0.010	−0.013
	beer	0.193	0.195	0.196	0.197	0.197	0.197
		−0.009	−0.007	−0.005	−0.004	−0.006	−0.008
	cider/alcop.	0.204	0.211	0.218	0.225	0.232	0.238
		−0.012	−0.009	−0.007	−0.006	−0.007	−0.009
SE	spirits	0.143	0.162	0.180	0.196	0.209	0.219
		−0.007	−0.006	−0.005	−0.007	−0.010	−0.015
	wine	0.193	0.170	0.147	0.125	0.104	0.085
		−0.009	−0.006	−0.005	−0.005	−0.006	−0.006
	beer	0.311	0.259	0.212	0.169	0.132	0.102
		−0.010	−0.007	−0.005	−0.005	−0.006	−0.006
	cider/alcop.	0.353	0.408	0.461	0.511	0.555	0.594
		−0.011	−0.008	−0.007	−0.008	−0.012	−0.016
UA	spirits	0.152	0.106	0.0723	0.049	0.033	0.022
		−0.010	−0.005	−0.003	−0.003	−0.003	−0.003
	wine	0.290	0.318	0.344	0.367	0.388	0.406
		−0.009	−0.007	−0.006	−0.007	−0.008	−0.011
	beer	0.207	0.206	0.201	0.193	0.184	0.175
		−0.008	−0.006	−0.005	−0.005	−0.006	−0.008
	cider/alcop.	0.351	0.370	0.383	0.391	0.395	0.397
		−0.010	−0.007	−0.006	−0.006	−0.009	−0.012

**Table A6:** Model predictions of beverage proportions in boys, 1999-2019 (margins, SE).

		1999	2003	2007	2011	2015	2019
BG	spirits	0.043	0.058	0.078	0.101	0.126	0.151
		−0.007	−0.007	−0.007	−0.005	−0.005	−0.009
	wine	0.0804	0.0974	0.115	0.133	0.147	0.157
		−0.009	−0.008	−0.006	−0.005	−0.005	−0.008
	beer	0.840	0.787	0.721	0.641	0.550	0.452
		−0.014	−0.013	−0.011	−0.008	−0.008	−0.012
	cider/alcop.	0.037	0.057	0.086	0.125	0.177	0.240
		−0.005	−0.005	−0.005	−0.005	−0.006	−0.011
HR	spirits	0.072	0.078	0.083	0.089	0.096	0.103
		−0.004	−0.003	−0.003	−0.003	−0.004	−0.006
	wine	0.308	0.311	0.314	0.317	0.320	0.323
		−0.008	−0.006	−0.005	−0.006	−0.008	−0.010
	beer	0.525	0.516	0.507	0.498	0.489	0.480
		−0.009	−0.007	−0.005	−0.005	−0.007	−0.009
	cider/alcop.	0.095	0.095	0.095	0.095	0.095	0.095
		−0.006	−0.004	−0.003	−0.004	−0.005	−0.006
CY	spirits	0.183	0.173	0.160	0.144	0.124	0.101
		−0.018	−0.012	−0.008	−0.006	−0.007	−0.009
	wine	0.168	0.160	0.150	0.137	0.119	0.098
		−0.019	−0.013	−0.007	−0.004	−0.006	−0.008
	beer	0.612	0.604	0.584	0.549	0.493	0.417
		−0.024	−0.017	−0.011	−0.009	−0.013	−0.021
	cider/alcop.	0.037	0.063	0.105	0.170	0.264	0.384
		−0.005	−0.007	−0.008	−0.010	−0.015	−0.024
CZ	spirits	0.097	0.105	0.113	0.120	0.125	0.126
		−0.004	−0.003	−0.003	−0.003	−0.004	−0.006
	wine	0.192	0.197	0.201	0.202	0.199	0.191
		−0.007	−0.005	−0.004	−0.005	−0.006	−0.008
	beer	0.696	0.671	0.641	0.604	0.557	0.499
		−0.008	−0.006	−0.005	−0.006	−0.007	−0.009
	cider/alcop.	0.016	0.027	0.045	0.074	0.118	0.184
		−0.002	−0.002	−0.003	−0.003	−0.004	−0.008
DK	spirits	0.051	0.062	0.075	0.090	0.108	0.128
		−0.009	−0.008	−0.007	−0.005	−0.005	−0.008
	wine	0.045	0.053	0.062	0.071	0.082	0.094
		−0.010	−0.009	−0.007	−0.005	−0.005	−0.009
	beer	0.441	0.439	0.435	0.428	0.420	0.409
		−0.040	−0.030	−0.021	−0.013	−0.010	−0.014
	cider/alcop.	0.463	0.447	0.429	0.411	0.390	0.369
		−0.043	−0.033	−0.023	−0.015	−0.011	−0.016
EE	spirits	0.093	0.102	0.111	0.120	0.129	0.138
		−0.008	−0.006	−0.004	−0.004	−0.005	−0.008
	wine	0.162	0.175	0.187	0.199	0.211	0.222
		−0.010	−0.008	−0.006	−0.005	−0.007	−0.011
	beer	0.502	0.462	0.423	0.384	0.347	0.311
		−0.014	−0.010	−0.007	−0.006	−0.008	−0.011
	cider/alcop.	0.243	0.261	0.279	0.297	0.313	0.329
		−0.010	−0.008	−0.006	−0.005	−0.007	−0.011
FI	spirits	0.100	0.102	0.103	0.105	0.107	0.109
		−0.006	−0.004	−0.003	−0.004	−0.005	−0.007
	wine	0.114	0.116	0.118	0.121	0.123	0.125
		−0.007	−0.005	−0.004	−0.004	−0.005	−0.007
	beer	0.496	0.494	0.492	0.490	0.488	0.486
		−0.009	−0.007	−0.006	−0.006	−0.009	−0.012
	cider/alcop.	0.290	0.288	0.286	0.284	0.282	0.280
		−0.009	−0.006	−0.005	−0.005	−0.007	−0.010
DE	spirits	0.072	0.073	0.073	0.074	0.074	0.074
		−0.006	−0.004	−0.003	−0.004	−0.006	−0.009
	wine	0.191	0.175	0.160	0.146	0.133	0.121
		−0.013	−0.008	−0.005	−0.006	−0.009	−0.012
	beer	0.515	0.540	0.565	0.589	0.613	0.636
		−0.015	−0.010	−0.007	−0.009	−0.014	−0.019
	cider/alcop.	0.223	0.212	0.202	0.191	0.180	0.170
		−0.012	−0.007	−0.005	−0.007	−0.011	−0.015
GR	spirits	0.190	0.193	0.196	0.198	0.201	0.203
		−0.009	−0.007	−0.005	−0.005	−0.006	−0.008
	wine	0.246	0.240	0.234	0.228	0.222	0.216
		−0.010	−0.007	−0.005	−0.004	−0.005	−0.007
	beer	0.397	0.406	0.415	0.424	0.433	0.442
		−0.014	−0.010	−0.007	−0.006	−0.008	−0.011
	cider/alcop.	0.167	0.161	0.155	0.150	0.145	0.139
		−0.011	−0.008	−0.005	−0.004	−0.004	−0.006
HU	spirits	0.170	0.160	0.149	0.139	0.129	0.118
		−0.008	−0.005	−0.004	−0.005	−0.006	−0.008
	wine	0.397	0.379	0.361	0.341	0.322	0.301
		−0.010	−0.007	−0.006	−0.006	−0.007	−0.009
	beer	0.359	0.374	0.389	0.402	0.414	0.424
		−0.010	−0.007	−0.006	−0.006	−0.007	−0.010
	cider/alcop.	0.074	0.086	0.101	0.117	0.135	0.156
		−0.005	−0.004	−0.004	−0.004	−0.005	−0.008
IS	spirits	0.138	0.147	0.157	0.166	0.176	0.185
		−0.008	−0.006	−0.006	−0.007	−0.011	−0.015
	wine	0.077	0.085	0.095	0.105	0.115	0.126
		−0.005	−0.004	−0.004	−0.005	−0.007	−0.011
	beer	0.636	0.609	0.582	0.555	0.526	0.498
		−0.010	−0.008	−0.007	−0.009	−0.012	−0.016
	cider/alcop.	0.149	0.158	0.166	0.174	0.182	0.190
		−0.006	−0.004	−0.005	−0.007	−0.009	−0.013
IT	spirits	0.180	0.147	0.119	0.094	0.073	0.055
		−0.009	−0.005	−0.003	−0.003	−0.003	−0.004
	wine	0.278	0.271	0.259	0.244	0.225	0.202
		−0.010	−0.007	−0.004	−0.004	−0.005	−0.008
	beer	0.461	0.471	0.474	0.469	0.454	0.429
		−0.010	−0.007	−0.005	−0.005	−0.007	−0.010
	cider/alcop.	0.0811	0.110	0.148	0.194	0.249	0.313
		−0.004	−0.004	−0.003	−0.004	−0.006	−0.011
LV	spirits	0.102	0.113	0.125	0.136	0.146	0.154
		−0.007	−0.006	−0.005	−0.005	−0.007	−0.010
	wine	0.177	0.175	0.171	0.166	0.158	0.149
		−0.009	−0.007	−0.006	−0.006	−0.007	−0.009
	beer	0.648	0.616	0.581	0.541	0.497	0.450
		−0.011	−0.009	−0.007	−0.008	−0.010	−0.012
	cider/alcop.	0.073	0.096	0.124	0.158	0.199	0.247
		−0.005	−0.005	−0.005	−0.005	−0.007	−0.011
LT	spirits	0.046	0.064	0.089	0.121	0.162	0.212
		−0.004	−0.004	−0.004	−0.004	−0.007	−0.012
	wine	0.146	0.164	0.182	0.199	0.214	0.225
		−0.008	−0.006	−0.005	−0.005	−0.008	−0.012
	beer	0.546	0.541	0.530	0.510	0.483	0.447
		−0.012	−0.008	−0.006	−0.006	−0.009	−0.013
	cider/alcop.	0.263	0.230	0.199	0.169	0.142	0.116
		−0.012	−0.008	−0.006	−0.005	−0.006	−0.007
MT	spirits	0.168	0.184	0.200	0.217	0.232	0.247
		−0.007	−0.006	−0.005	−0.006	−0.008	−0.011
	wine	0.354	0.336	0.317	0.297	0.276	0.254
		−0.011	−0.009	−0.007	−0.007	−0.008	−0.010
	beer	0.413	0.399	0.383	0.365	0.345	0.323
		−0.009	−0.007	−0.006	−0.007	−0.009	−0.010
	cider/alcop.	0.065	0.081	0.099	0.121	0.147	0.177
		−0.004	−0.004	−0.003	−0.004	−0.006	−0.009
NL	spirits	0.204	0.159	0.121	0.089	0.063	0.043
		−0.021	−0.011	−0.006	−0.005	−0.006	−0.006
	wine	0.073	0.078	0.082	0.083	0.081	0.076
		−0.010	−0.007	−0.005	−0.004	−0.006	−0.008
	beer	0.673	0.683	0.674	0.645	0.594	0.524
		−0.022	−0.014	−0.010	−0.009	−0.010	−0.014
	cider/alcop.	0.051	0.080	0.123	0.183	0.262	0.358
		−0.005	−0.006	−0.007	−0.007	−0.010	−0.016
NO	spirits	0.094	0.110	0.129	0.150	0.174	0.199
		−0.010	−0.009	−0.008	−0.007	−0.009	−0.013
	wine	0.111	0.106	0.101	0.096	0.090	0.085
		−0.010	−0.007	−0.005	−0.005	−0.007	−0.008
	beer	0.339	0.354	0.368	0.380	0.391	0.399
		−0.016	−0.012	−0.010	−0.010	−0.013	−0.017
	cider/alcop.	0.457	0.429	0.402	0.374	0.345	0.317
		−0.016	−0.012	−0.009	−0.009	−0.011	−0.014
PO	spirits	0.096	0.105	0.115	0.125	0.135	0.146
		−0.005	−0.004	−0.003	−0.003	−0.004	−0.006
	wine	0.160	0.159	0.158	0.156	0.153	0.150
		−0.007	−0.005	−0.004	−0.004	−0.005	−0.006
	beer	0.727	0.712	0.696	0.678	0.658	0.634
		−0.009	−0.007	−0.006	−0.005	−0.007	−0.010
	cider/alcop.	0.018	0.024	0.032	0.042	0.054	0.071
		−0.003	−0.003	−0.002	−0.002	−0.003	−0.006
PT	spirits	0.246	0.247	0.247	0.247	0.246	0.244
		−0.009	−0.007	−0.006	−0.005	−0.007	−0.009
	wine	0.0897	0.100	0.112	0.124	0.138	0.153
		−0.007	−0.005	−0.004	−0.004	−0.006	−0.008
	beer	0.515	0.498	0.481	0.464	0.446	0.428
		−0.011	−0.008	−0.007	−0.007	−0.008	−0.011
	cider/alcop.	0.149	0.155	0.160	0.166	0.171	0.175
		−0.007	−0.005	−0.005	−0.005	−0.006	−0.008
RO	spirits	0.016	0.027	0.040	0.056	0.076	0.101
		−0.002	−0.002	−0.003	−0.003	−0.004	−0.006
	wine	0.146	0.186	0.216	0.237	0.249	0.256
		−0.007	−0.006	−0.005	−0.005	−0.006	−0.008
	beer	0.440	0.537	0.599	0.628	0.633	0.622
		−0.017	−0.011	−0.008	−0.007	−0.008	−0.010
	cider/alcop.	0.398	0.251	0.145	0.0788	0.0411	0.0209
		−0.020	−0.010	−0.005	−0.004	−0.004	−0.003
SK	spirits	0.163	0.174	0.185	0.194	0.201	0.204
		−0.007	−0.006	−0.005	−0.005	−0.006	−0.009
	wine	0.401	0.365	0.328	0.293	0.257	0.222
		−0.013	−0.009	−0.006	−0.006	−0.008	−0.010
	beer	0.422	0.439	0.453	0.462	0.465	0.459
		−0.015	−0.011	−0.007	−0.007	−0.009	−0.012
	cider/alcop.	0.014	0.022	0.034	0.052	0.078	0.115
		−0.002	−0.002	−0.003	−0.003	−0.004	−0.007
SI	spirits	0.063	0.0745	0.088	0.103	0.121	0.140
		−0.005	−0.004	−0.004	−0.005	−0.007	−0.010
	wine	0.373	0.351	0.330	0.308	0.287	0.266
		−0.011	−0.008	−0.007	−0.007	−0.009	−0.011
	beer	0.410	0.417	0.422	0.425	0.427	0.426
		−0.011	−0.008	−0.006	−0.006	−0.008	−0.011
	cider/alcop.	0.153	0.157	0.161	0.164	0.166	0.168
		−0.007	−0.006	−0.005	−0.004	−0.005	−0.006
SE	spirits	0.127	0.140	0.154	0.167	0.181	0.194
		−0.006	−0.005	−0.004	−0.006	−0.009	−0.012
	wine	0.101	0.095	0.089	0.083	0.076	0.070
		−0.007	−0.005	−0.004	−0.005	−0.006	−0.007
	beer	0.524	0.486	0.448	0.410	0.371	0.334
		−0.010	−0.007	−0.006	−0.007	−0.010	−0.013
	cider/alcop.	0.249	0.278	0.309	0.340	0.372	0.403
		−0.008	−0.006	−0.005	−0.006	−0.008	−0.011
UA	spirits	0.195	0.143	0.102	0.072	0.050	0.035
		−0.010	−0.005	−0.004	−0.004	−0.004	−0.004
	wine	0.181	0.203	0.222	0.239	0.254	0.268
		−0.008	−0.007	−0.006	−0.006	−0.007	−0.009
	beer	0.425	0.432	0.430	0.421	0.408	0.391
		−0.011	−0.008	−0.007	−0.007	−0.009	−0.011
	cider/alcop.	0.198	0.223	0.246	0.268	0.288	0.306
		−0.009	−0.007	−0.005	−0.005	−0.008	−0.011
